# NEOnatal Central-venous Line Observational study on Thrombosis (NEOCLOT): evaluation of a national guideline on management of neonatal catheter-related thrombosis

**DOI:** 10.1186/s12887-018-1000-7

**Published:** 2018-02-23

**Authors:** Jeanine J. Sol, Moniek van de Loo, Marit Boerma, Klasien A. Bergman, Albertine E. Donker, Mark A. H. B. M. van der Hoeven, Christiaan V. Hulzebos, Ronny Knol, K. Djien Liem, Richard A. van Lingen, Enrico Lopriore, Monique H. Suijker, Daniel C. Vijlbrief, Remco Visser, Margreet A. Veening, Mirjam M. van Weissenbruch, C. Heleen van Ommen

**Affiliations:** 1 Department of Pediatrics, Groene Hart Hospital, Gouda, the Netherlands; 2 Neonatal Intensive Care Unit, Sophia Children’s Hospital Erasmus MC, Rotterdam, the Netherlands; 3 Neonatal Intensive Care Unit, Emma Children’s Hospital AMC, Amsterdam, the Netherlands; 4 Department of Pediatric Hematology, Sophia Children’s Hospital Erasmus MC, Postbus 2060, 3015 CN Rotterdam, the Netherlands; 5 Neonatal Intensive Care Unit, Beatrix Children’s Hospital UMCG, Groningen, the Netherlands; 6 Department of Pediatric Hematology, Maxima Medisch Centrum, Veldhoven, the Netherlands; 7 Neonatal Intensive Care Unit, MUMC, Maastricht, the Netherlands; 8 Neonatal Intensive Care Unit, Neonatal Intensive Care Unit, Beatrix Children’s Hospital UMCG, Groningen, the Netherlands; 9 Neonatal Intensive Care Unit, Amalia Children’s Hospital Radboud UMC, Nijmegen, the Netherlands; 10 Neonatal Intensive Care Unit, Isala Clinics, Zwolle, the Netherlands; 11 Neonatal Intensive Care Unit, Willem-Alexander Hospital LUMC, Leiden, the Netherlands; 12 Department of Pediatric Hematology, Emma Children’s Hospital AMC, Amsterdam, the Netherlands; 13 Neonatal Intensive Care Unit, Wilhelmina Children’s Hospital UMCU, Utrecht, the Netherlands; 14 Department of Pediatric Hematology, VUMC, Amsterdam, the Netherlands; 15 Neonatal Intensive Care Unit, VUMC, Amsterdam, the Netherlands

**Keywords:** Neonate, Catheter, Thrombosis, Antithrombotic therapy, Observational

## Abstract

**Background:**

In critically ill (preterm) neonates, central venous catheters (CVCs) are increasingly used for administration of medication or parenteral nutrition. A serious complication, however, is the development of catheter-related thrombosis (CVC-thrombosis), which may resolve by itself or cause severe complications. Due to lack of evidence, management of neonatal CVC-thrombosis varies among neonatal intensive care units (NICUs). In the Netherlands an expert-based national management guideline has been developed which is implemented in all 10 NICUs in 2014.

**Methods:**

The NEOCLOT study is a multicentre prospective observational cohort study, including 150 preterm and term infants (0-6 months) admitted to one of the 10 NICUs, developing CVC-thrombosis. Patient characteristics, thrombosis characteristics, risk factors, treatment strategies and outcome measures will be collected in a web-based database. Management of CVC-thrombosis will be performed as recommended in the protocol. Violations of the protocol will be noted. Primary outcome measures are a composite efficacy outcome consisting of death due to CVC-thrombosis and recurrent thrombosis, and a safety outcome consisting of the incidence of major bleedings during therapy. Secondary outcomes include individual components of primary efficacy outcome, clinically relevant non-major and minor bleedings and the frequency of risk factors, protocol variations, residual thrombosis and post thrombotic syndrome.

**Discussion:**

The NEOCLOT study will evaluate the efficacy and safety of the new, national, neonatal CVC-thrombosis guideline. Furthermore, risk factors as well as long-term consequences of CVC-thrombosis will be analysed.

**Trial registration:**

Trial registration: Nederlands Trial Register NTR4336. Registered 24 December 2013.

## Background

In critically ill (preterm) neonates, central venous catheters (CVCs) are increasingly used for administering medication or parenteral nutrition. These catheters are inserted in umbilical veins, major central veins or in smaller peripheral veins. CVCs are one of the stepping stones in improvement of care for critically ill neonates. However, one of the complications associated with CVC usage is venous thrombosis. The prevalence of neonatal CVC-related thrombosis (CVC-thrombosis) varies from 0.7% to 67% and is dependent on the type of catheter inserted, the diagnostic tests used, the study method and the index of suspicion of thrombosis [1–3].

Evidence in literature on optimal management of neonates with CVC-thrombosis is lacking [4]. Only case-series and case reports are available. Therapeutic options include 1) a “wait and see” policy (an expectative policy monitored with ultrasonography), 2) anticoagulant treatment, 3) thrombolysis, and 4) thrombectomy [4].

### The “wait and see” policy

The “wait and see” policy might be an option as spontaneous regression of CVC-thrombosis has been described. Butler-O’Hara et al. reported spontaneous regression in 13 of 24 children with umbilical venous catheter thrombosis after a median duration of 28 days, without anticoagulation [5]. Small retrospective studies confirmed this observation [3, 6]. In addition, Kim et al. prospectively studied the incidence of neonatal portal venous thrombosis associated with catheterization of the umbilical vein. Ultrasonography demonstrated asymptomatic portal venous thrombosis in 43 of 100 neonates. Follow-up ultrasonography showed complete or partial resolution in 20 (56%) of 36 neonates without treatment. A significant negative relationship was found between the initial size of thrombosis and spontaneous clot resolution [7].

On the other hand, CVC-thrombosis may increase in size and cause potential life-threatening acute and/or chronic complications [8]. CVC-thrombosis in the right atrium may lead to tricuspid valve obstruction, pulmonary embolism with severe respiratory insufficiency and heart failure. Cerebral embolism via a patent foramen ovale may cause stroke [9]. The exact prevalence of these complications remains unknown. However, the potential life threatening character of these complications warrant the use of antithrombotic measures, including anticoagulants, thrombolysis and thrombectomy.

### Anticoagulant treatment

Little is known about the efficacy and safety of anticoagulant and thrombolytic agents in neonates. To the best of our knowledge, no randomized controlled trials have been conducted to date. Low molecular weight heparin (LMWH) is the most prescribed anticoagulant agent in neonates [4, 10]. In adults, LMWH is as effective as unfractionated heparin (UFH) with decreased risk of bleeding complications and heparin-induced thrombocytopenia [11]. The pharmacokinetics of LMWH are more predictable than those of UFH, resulting in less frequent dose adjustments and monitoring to achieve the therapeutic range. In addition, LMWH can be administered subcutaneously, once or twice daily. In children, therefore, LMWH as enoxaparin has already become the agent of choice in treatment of thrombosis [12].

Malowany et al. reviewed all studies between 1980 and 2007 in which enoxaparin was used to treat neonates. Enoxaparin was administered to 240 neonates (53 preterm, 61 term and 126 neonates with unknown gestational age) with venous thrombosis. Preterm neonates required a higher dose of enoxaparin than term neonates. A starting dose of 1.7 mg/kg enoxaparin per 12 h in term and 2 mg/kg enoxaparin per 12 h in preterm neonates is suggested: Eighty-six of 119 neonates (72%) demonstrated complete or partial resolution of thrombosis. The overall major bleeding rate was 4% (9 of 217 neonates) [11]. In later studies, reported bleeding rate raged from 0 to 4% [13–16].

### Thrombolytic treatment

Three thrombolytic agents are available, i.e. streptokinase, urokinase and recombinant tissue plasminogen activator (r-tPA). In contrast to streptokinase and urokinase, r-tPA has an increased affinity for fibrin-bound plasminogen, which theoretically makes the drug more effective near the thrombus than streptokinase or urokinase, whilst also potentially lowering the risk of bleeding. However, no in vivo studies have been carried out to support the theorised advantage of r-tPA. In general, r-tPA is most frequently used for thrombolysis in children. In a literature review, Torres-Valdivieso et al. reviewed literature and analyzed 98 neonates treated with varying doses of r-tPA [17]. The clot completely resolved in 70%, it partially disappeared in 20% and remained unaffected in 10% of the patients. However, the complication rate was high: 4% of neonates died as a result of major bleeding, 10% experienced intraventricular bleeding, 2% suffered pulmonary bleeding, 1% had kidney bleeding and 5% had minor bleeding [17]. The precise r-tPA dosage for thrombolysis in neonates is unknown. In vitro studies have demonstrated that neonates are slow responders to fibrinolytic drugs, which might be explained by lower plasminogen plasma values than in adults. Adding plasma has been shown to accelerate the fibrinolytic process [18].

### Thrombectomy

Thrombectomy is the fourth therapeutic option in neonatal thrombosis. However, in neonates thrombectomy is usually impossible due to the small calibre of the vessels. Additionally, re-occlusion occurs frequently. Surgical thrombectomy of the thrombus in the right atrium is a highly invasive and dangerous procedure. Only a few case reports are available in neonates [6, 8].

### National guideline

How to choose between the four therapeutic options in neonates with thrombosis? The American College of Chest Physicians (ACCP) evidence-based guideline of 2012 recommends either to treat neonatal CVC-thrombosis with anticoagulants and/or to monitor it with ultrasonography [4]. Anticoagulant agents should be administered if extension of thrombosis occurs. The ACCP guideline discourages thrombolytic therapy for neonatal CVC-thrombosis unless major vein occlusion is causing critical comprise of organs or limbs. Yang et al. tried to outline high-risk right atrial thrombosis in a literature review of 122 neonates and children (41% were preterm infants). They defined right atrial thrombosis as high-risk if thrombosis was large, pedunculated, mobile, or snake-shaped and mobile. A significant difference in mortality was found between the high-risk group (16,7%; 3 of 18) and the low-risk group (0%; 0 of 32) [8].

**Fig. 1:**
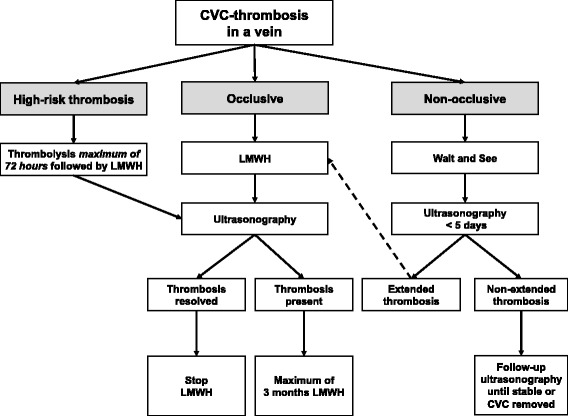
CVC-thrombosis in a blood vein

**Fig. 2:**
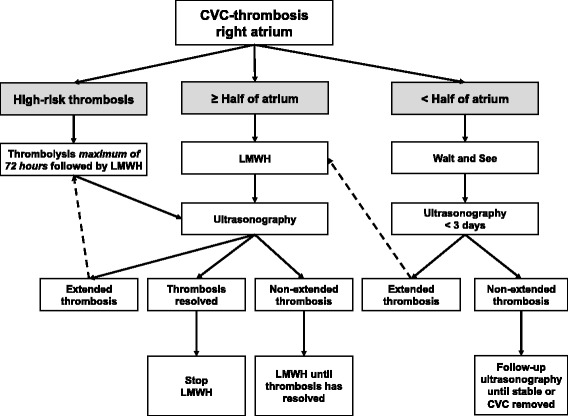
CVC-thrombosis in the right atrium

The NEOCLOT working group has refined the ACCP recommendations into a more detailed guideline based on the scarce data and expert opinion in order to standardize treatment of neonatal CVC-thrombosis nationally. For the management of neonatal CVC-thrombosis a distinction was made between CVC-thrombosis located in a blood vein (non-occlusive versus occlusive) and CVC-thrombosis located in the right atrium. Furthermore, high-risk (life-threatening) thrombosis was defined (Figs. 1 and 2). In the NEOCLOT study, evaluation of this new guideline will be performed. This article describes the study protocol of the NEOCLOT study.

## Methods/design

### Aim of the study

The primary aim of the NEOCLOT study is to evaluate the efficacy and safety of the management of CVC-thrombosis in neonates as advised in the national guideline for neonatal CVC-thrombosis. Secondary aims include the evaluation of risk factors for neonatal CVC-thrombosis, the adherence to the guideline and the frequency of chronic complications of neonatal CVC-thrombosis after 1 year of follow-up.

### Study design and setting

The NEOCLOT study is a multi-center prospective observational cohort study conducted in all 10 neonatal intensive care units (NICUs) in the Netherlands. The inclusion period will be at least 5 years. All patients will be followed for a minimum of 1 year. The Medical Ethics Review Committee confirmed that official approval of this study was not required as the Medical Research Involving Human Subjects Act did not apply to the NEOCLOT study. (#14.17.0121).

### Study population

#### Inclusion criteria

All preterm and term infants (0-6 months) admitted on one of the NICUs with CVC-thrombosis will be included.

#### Diagnosis of CVC-thrombosis

Symptoms of neonatal CVC-thrombosis include swelling, erythema, skin discoloration, increased warmth, pain, and/or tenderness of the affected arm or leg, venous distension, presence of subcutaneous collateral veins, superior vena cava syndrome, loss of central venous catheter patency, prolonged catheter-related septicaemia, unexplained thrombocytopenia, arrhythmia and hemodynamic instability [19]. Symptomatic CVC-thrombosis has to be confirmed by Doppler ultrasonography. CVC-thrombosis is diagnosed via ultrasonography if a non-compressible segment of a vein, absence of flow, or an echogenic intraluminal thrombus is present.

CVC-thrombosis in a vein is defined as a non-obstructive clot if blood flow is still present and as an obstructive clot if blood flow is absent. High-risk CVC-thrombosis in veins is defined as thrombosis which compromises an organ or limb. High-risk thrombosis in the right atrium is defined as thrombosis, which 1) restricts the outflow from the right atrium via the tricuspid valve, 2) extends via the tricuspid valve or patent foramen ovale, 3) causes severe arrhythmias, 4) causes hemodynamic instability, 5) is pedunculated, mobile, or snake-shaped and mobile, and 6) grows despite adequate therapeutic heparin levels.

### Treatment of patients

In all neonates with CVC-thrombosis, it is advised to remove the CVC, if possible.

Treatment of CVC-thrombosis is divided into treatment of CVC-thrombosis in veins and CVC-thrombosis in the right atrium. In both scenarios, it is necessary to establish whether the thrombosis is deemed high-risk. In addition, the risks and benefits of all treatment options versus risks of ongoing thrombosis should be considered in each neonate before treatment is started. Relative contraindications for anticoagulation and thrombolysis include invasive surgical procedure(s) in the preceding 10 days, intracranial bleeding in the preceding 10 days, invasive surgical procedure(s) scheduled within 3 days, active bleeding, severe asphyxia, very preterm neonates (< 28 weeks) with high risk of intraventricular haemorrhage and severe thrombocytopenia.

#### CVC-thrombosis in a blood vein

Figure 1 shows the consensus-based algorithm regarding the proposed policy to CVC-thrombosis in a blood vein. High-risk CVC-thrombosis should be treated with thrombolytic therapy followed by anticoagulant therapy for at least 4 to 6 weeks. During thrombolysis ultrasonography will be performed once daily (see Table 1).

**Table 1:** Thrombolytic therapy

Thrombolytic agent	Therapeutic dose	Monitoring
r-TPA	Start: 0.1 mg/kg/h iv Continuous infusion for longer periods with increasing doses if no improvement Max. dose: 0.5 mg/kg/h iv	Check CBC, APTT, PT, fibrinogen, D-dimers daily Exclude ICH by US daily Transfuse with FFP daily Maintain fibrinogen > 1.0 g/L and platelets > 50 × 10^9^/L Check thrombus resolution once to twice daily

*Abbreviations*: *r-TPA* recombinant tissue plasminogen activator, *h* hour, *iv* intravenously, *CBC* complete blood count, *APTT* activated partial thromboplastin time, *PT* prothrombin time, *ICH* intracranial hemorrhage, *US* ultrasound, *FFP* Fresh frozen plasma, *max* maximum

After cessation of thrombolysis, LMWH should always be started. After 4 to 6 weeks, ultrasonography will be performed. If the clot has disappeared, anticoagulation will be stopped.

For non-obstructive CVC-thrombosis, a “wait and see” policy is recommended, with Doppler ultrasonography follow-up within 5 days, depending on the size of thrombosis. If the size of thrombosis increases, anticoagulant therapy should be started. In all neonates with obstructive CVC-thrombosis and without indication for thrombolysis, anticoagulant therapy (LMWH) should be started immediately.

#### CVC-thrombosis in the right atrium

Figure 2 shows the consensus-based algorithm regarding the proposed policy to CVC-thrombosis in the right atrium. If thrombosis in the right atrium is defined as high-risk and there are no contraindications for thrombolytic therapy, thrombolysis should be administrated as soon as possible. After cessation of thrombolysis, LMWH should always be started. After 4 to 6 weeks of LMWH, ultrasonography will be performed. If the clot has disappeared, anticoagulation will be stopped.

A “wait and see” policy is recommended for CVC-thrombosis in the right atrium obstructing less than half of the atrium and without indication for thrombolysis. Echocardiographic follow-up of these thrombi should be performed every 1 to 3 days, depending on the size of thrombosis. If thrombosis extends during the “wait and see” policy, anticoagulant therapy should be started. If CVC-thrombosis fills more than half of the right atrium and has no indication for thrombolytic therapy, anticoagulant therapy (LMWH) should be started immediately.

#### Anticoagulant and thrombolytic therapy

The working group prefers r-tPA above urokinase and streptokinase due to assumed increased affinity for fibrin-bound plasminogen. Table 1 shows the protocol for thrombolysis with r-tPA.

LMWH is preferred above UFH due to reduced need of monitoring, potential decreased risk of bleeding and the greater customisability in the Netherlands. Table 2 shows the LMWH protocol.

**Table 2:** Anticoagulant therapy [4, 11, 13]

LMWH	Therapeutic dose	Monitoring
Nadroparin 0 – 2 m Enoxaparin 0 – 2 m Dalteparin 0 – 2 m Tinzaparin 0 – 2 m	120-150 U/kg/12 h sc 1,7 mg/kg/12 h sc in preterm neonates 1,5 mg/kg/12 h sc in term neonates 150 U/kg/12 h sc 275 U/kg/24 h sc	Check anti-FXa level 4 h after 2nd dose; Target anti-FXa level: 0.5–1.0 U/mL Check platelets regularly

*Abbreviations*: *LMWH* low-moleculair-weight heparin, *h* hour, *m* months, *sc* subcutaneously

When LMWH is administered via a subcutaneous port (Insuflon©), it is important to check the injection site and to change the port at regular intervals, especially in neonates with little subcutaneous fat. Alternatively, one can refrain from using such a port. Platelet transfusions are not encouraged when thrombocytopenia is present as these transfusions may contribute to extension of thrombosis. Alternatively, dosage of LMWH may be reduced to prophylactic dose, depending on size of thrombosis, risk of embolization and duration of previous treatment period. The maximum duration of antithrombotic therapy in neonatal CVC-thrombosis is 3 months. If at an earlier stage ultrasonography shows that thrombosis has resolved, antithrombotic therapy can be stopped. Ideally these ultrasounds are performed at 6 weeks. However, when a child is discharged before 6 weeks, an ultrasound will be performed earlier.

### Outcome measures

#### Outcomes of primary aims

The primary efficacy outcome for the NEOCLOT study is a composite outcome consisting of recurrent thrombosis and death due to CVC-thrombosis after start of management of CVC-thrombosis. The primary safety outcome is the incidence of major bleedings during thrombolytic and anticoagulant therapy.

Major bleeding is defined as reported by Mitchell et al.: 1) fatal bleeding, 2) clinically overt bleeding associated with a decrease in hemoglobin of at least 20 g/L (i.e., 2 g/dL or 1.24 mmol/L) in a 24-h period, (3) bleeding that is retroperitoneal or pulmonary, or (4) bleeding that requires surgical intervention in an operating room [20]. Intracranial bleeding is categorized major bleeding as defined by Curley et al. in the Planet-2 study: Intraventricular haemorrhage (IVH) (H1, H2 or H3) with ventricular dilatation, IVH (H1, H2, H3) with parenchymal extension, any evolution of intracranial haemorrhage from IVH or germinal layer heamorrhage to IVH with ventricular dilatation or IVH with parenchymal extension [21].

The secondary efficacy outcomes are the individual components of the primary outcome, i.e. death due to CVC-thrombosis and recurrent thrombosis and all-cause mortality. The secondary safety outcomes include clinically relevant non-major bleeding (CRNMB) and minor bleedings during fibrinolytic and anticoagulant therapy as defined by Mitchell et al. [20]. All intracranial bleedings which are not defined as major bleeding will be categorized as non-major intracranial bleeding. CRNMB is a composite of (1) overt bleeding for which a blood product is administered and not directly attributable to the patient’s underlying medical condition and (2) bleeding that requires medical or surgical intervention to restore hemostasis, other than in an operating room. Minor bleeding is defined as any overt or macroscopic evidence of bleeding that does not fulfil the above criteria for either major bleeding or CRNMB.

#### Outcomes of secondary aims

Outcomes of the secondary aims consist of frequency of risk factors for CVC-thrombosis, frequency of protocol variations and frequency and severity of long-term consequences after 1, 2 and 5 years, including post thrombotic syndrome (PTS) and residual thrombosis.

### Data collection

Data from all neonates with CVC-thrombosis will be added to the Good Clinical Practice proof web-based NEOCLOT database. This database is only accessible for the participating investigators. Security is guaranteed with login names, login codes and encrypted data transfer. Data of all patients will be coded and the key to this coding is only known to the local investigator.

The following data will be collected in the web-based NEOCLOT database:

- Baseline characteristics: gestational age, birth weight, gender, Apgar score at 5 min, mechanical ventilation at time of diagnosis
- Characteristics of CVC-thrombosis: date, location, diagnostic method, symptoms, size, occlusive or non-occlusive, high-risk or low-risk thrombosis
- Potential risk factors for CVC-thrombosis: type of CVC, size of CVC, number of attempted CVC insertions, number of lumens, place of insertion of CVC, location of catheter tip, CVC-days, CVC-infection according to the National Healthcare Safety Network criteria [22], suspected CVC-infection, polycythaemia (venous haematocrit above 0.65 L/L), the presence of disseminated intravascular coagulation [23], shock (hypotension, needing therapy), congenital heart disease, recent surgery, family history of thrombophilia, and maternal problems including maternal diabetes and antiphospholipid syndrome.
- Treatment of CVC-thrombosis: applied policy, catheter removal, duration and dosages of thrombolytic and anticoagulant therapy, effect of applied policy, and complications of therapy, including bleeding complications.
- Follow-up: death due to thrombosis or other reason, pulmonary embolism, stroke, recurrent thrombosis, and residual thrombosis after end of therapy. Residual thrombosis will be determined by using Doppler ultrasonography until thrombosis has disappeared. PTS will be assessed at the NICU outpatient follow-up clinic after 1, 2 and 5 years according to the modified Villalta score [24]. At 5-years follow-up the new developed CAPTSureTM will be used, as well [25].

### Statistical analysis

#### Sample size calculation

The most important safety outcome of this prospective observational study is major bleeding. In the literature the mean prevalence of major bleeding in neonates with antithrombotic agents is about 10%. With this national guideline we expect the effect on the outcome of major bleeding in all neonates, to decline from 10 to 5%. Given that we have 150 neonates, the 95% CI will be about 2.5 to 9.9%, which means that we have a large probability of a significant difference with the literature. The formula for the CI is based on the binomial distribution for independent cases.

Baseline data will be analysed by descriptive statistics. Data will be presented as mean and standard deviations or medians and ranges depending on their distribution. The proportion of patients who developed the primary and secondary outcomes will be shown. Continuous variables will be analysed using Student’s *t* test or Mann-Whitney test. Categorical data will be analysed using chi-square test or Fisher exact test. The proportion of patients with various risk factors for CVC-thrombosis will be calculated. The significance level is set at *p* < 0.05. Data will be analysed using PASW Statistics (SPSS) 20.0.

## Discussion

Advances in medical and surgical management has improved survival of sick (preterm) neonates, but has caused an increased incidence of thrombo-embolic complications. Lack of prospective clinical trials of antithrombotic treatment in (preterm) neonates leads to extrapolation of results of adult management studies to children. However, extrapolation of adult results to (preterm) neonates is difficult due to differences between neonatal and adult hemostasis and the presence of severe underlying medical conditions increasing the risk of bleeding complications in these vulnerable infants [26].

Furthermore, natural history of neonatal thrombosis seems to differ from that of adult thrombosis, as about 50% of neonatal thrombi appears to vanish without anticoagulant therapy. Determination of the natural history of neonatal thrombosis and identification of these “non-risky” thrombi is important to safely withhold anticoagulation in future patients.

As result of the national guideline, antithrombotic treatment of neonatal CVC-thrombosis has become identical on all NICUs in the Netherlands. Prospective collection of the neonates treated according to the protocol will enable evaluation of the used management strategy and generate data that can be used in follow-up treatment studies. For example, the NEOCLOT study allows investigating the natural history of specific neonatal catheter-related clots. In the current guideline “non-risky” thrombi were defined as non-obstructive thrombi in veins and thrombi filling less than 50% of the right atrium. Wait and see policy is applied to these thrombi. Results of the NEOCLOT study will show whether it will be safe to withhold anticoagulation in neonates with these thrombi.

## Abbreviations

ACCP: American College of Chest Physicians
CRNMB: Clinically relevant non-major bleeding
CVC: Central venous catheter
IVH: Intraventricular haemorrhage
LMWH: Low-molecular-weight heparin
NICU: Neonatal Intensive Care Unit
PTS: Post thrombotic syndrome
r-TPA: Recombinant tissue plasminogen activator
UFH: Unfractionated heparin
